# Supplementary material for: Remodeling of active endothelial enhancers is associated with aberrant gene-regulatory networks in pulmonary arterial hypertension
Source: Nat Commun. 2020 Apr 3;11:1673. doi: 10.1038/s41467-020-15463-x (PMC7125148; doi:10.1038/s41467-020-15463-x)
Supplement: Supplementary file 1 — Supplementary Information [file 41467_2020_15463_MOESM1_ESM.pdf]

Remodeling of active endothelial enhancers is associated with aberrant gene-regulatory networks in pulmonary arterial hypertension

Reyes-Palomares et al.

Supplementary Information

1. Supplementary Methods
2. Supplementary Table
3. Supplementary Figures
4. Supplementary References

1. Supplementary Methods

Comparison of IPAH and HPAH reveals high similarity between the two

When only considering the HPAH cases we found a reduced number of differentially H3K27ac marked regions when compared to healthy controls, likely due to the low number of patients (2 patients vs 7 controls, 25/71 at FDR 5/10%). Reassuringly, however, when comparing the fold-change signal between only IPAH vs controls and only HPAH vs controls we do find a significant correlation (R=0.59; **Supplementary Figure 1F**), indicating that it is the low number of individuals in the HPAH cases that leads to the reduced number of significant changes. In contrast, when performing the same analyses on the enhancer mark H3K4me1, the promoter mark H3K4me3 or RNA expression, the differences were not significant.

2. Supplementary Table

Samples	H3K4me3	H3K27ac	H3K4me1
**CTRL-2**	48672	80607	166317
**CTRL-3**	47601	123690	154360
**CTRL-4**	41424	97398	142448
**CTRL-5**	45339	89146	142288
**CTRL-6**	53352	86508	125699
**CTRL-7**	48068	102182	117485
**CTRL-8**	40319	NA	114522
**CTRL-9**	33643	101925	110622
**CTRL-10**	44384	NA	134415
**PAH-1**	50623	80737	126837
**PAH-2**	50838	89623	148215
**PAH-3**	43748	141866	172011
**PAH-4**	46236	79099	156604
**PAH-5**	45530	88683	115388
**PAH-6**	50401	76838	138093
**PAH-7**	41609	96325	142189
**PAH-8**	54513	81895	153209
**PAH-9**	33180	123988	114165
**PAH-10**	46570	81992	129094

Supplementary Table 1. Total ChIP peaks identified from all patient and control samples in pulmonary arterial endothelial cells for H3K4me3, H3K27ac and H3K4me1.

3. Supplementary Figures

Supplementary Figure 1

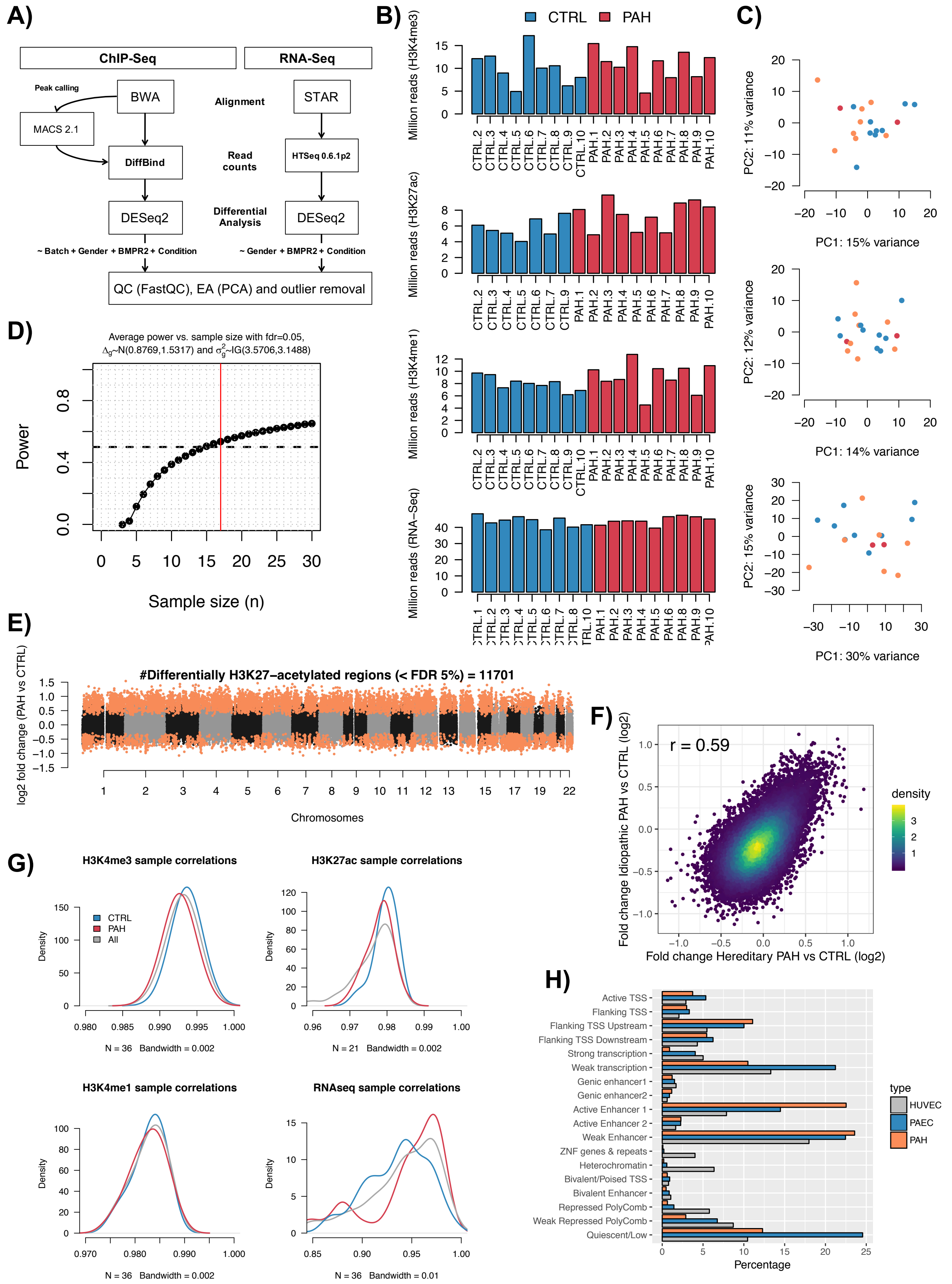

Supplementary Figure 1. Histone modification and gene expression changes in PAH and functional analysis of differentially modified H3K27ac regions. **A)** Schematic overview of the processing pipeline employed for ChIP-Seq and RNA-Seq data. **B)** The number of read counts per sample is shown. **C)** Principal component analysis for the top 1000 most variable regions of H3K4me3 (top), H3K4me1 (middle) and RNA (bottom) are shown. Individuals are colored by disease status: idiopathic PAH (orange), hereditary PAH (red) and control (blue). **D)** Power analysis of the differential H3K27ac analysis. R package ssizeRNA (version 1.3.1)[1] was used to estimate power based on simulations (negative binomial distribution assuming a general linear model and allowing for unequal dispersion between conditions). The result indicates that with a sample size of 19, which we used in the study, we are already in the plateauing range and approximating 60% power at an FDR of 0.05. **E)** Fold-change distributions for the differential H3K27ac analysis PAH vs controls are shown as Manhattan plot. Orange dots represent significant differentially modified regions (< FDR 5%). **F)** Log-ratio vs mean expression (MA plot) are shown for H3K4me3 (left) and H3K4me1 (right) signal between patients and controls; red dots represent significantly modified regions (< FDR 5%). **G)** Distribution of pairwise correlation coefficients for comparing the signal across peaks or genes between pairs of individuals. Correlations are shown for pairs of patients (red), pairs of controls (blue) and pairs between patients and control (grey). RNA is least correlated between any pair, suggesting heterogeneity as one reason for the lack in differential expression signal. **H)** Distribution of chromatin states of H3K27ac regions detected in PAECs (blue bars, representing 263,910 genomic features) and in differentially modified regions (orange bars, representing 31,084 genomic features). Chromatin states were obtained from human umbilical vein endothelial cell (HUVEC; grey bars, representing 515,807 genomic features) from the Roadmap Project12 (file: E122_18_core_K27ac_dense.bed).

Supplementary Figure 2

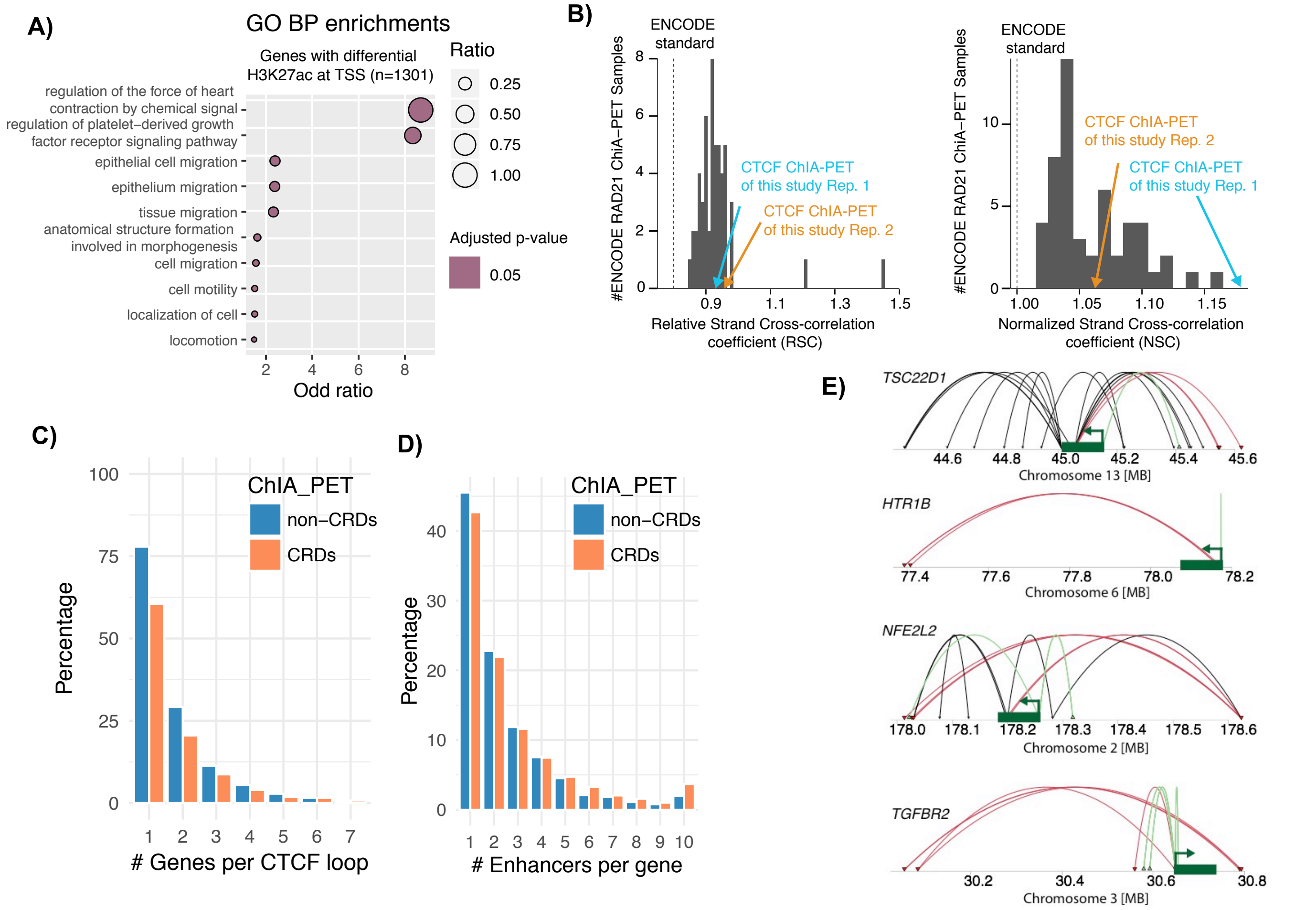

Supplementary Figure 2. CRDs and co-regulatory interactions between genes and H3K27ac signals. **A)** GO enrichment of genes that have differentially H3K27acetylated peak in their promoters. Source data are provided as a Source Data file. **B)** Quality control measures for our CTCF-ChIA-PET data in comparison with the ENCODE RAD21 ChIA-PET data. RSC and NSC represent the relative and normalized strand cross correlation coefficient, respectively[2]. The ENCODE standard for ChIA-PET is indicated as a vertical dashed line. All our replicates are well above the ENCODE standard and within the distribution of the ENCODE ChIA-PET for RAD21. **C)** Number of genes per CTCF-loop in CRD vs non-CRD loops. **D)** Number of enhancers per gene when using CRD (orange) vs non-CRD loops (blue). **E)** Examples of ChIA-PET data is shown for four genes also shown in Figure 1F. CRD-ChIA PET loops are shown in red, correlation-based enhancer-gene interactions are shown in green and non-CRD ChIA-PET loops are shown in grey.

Supplementary Figure 3

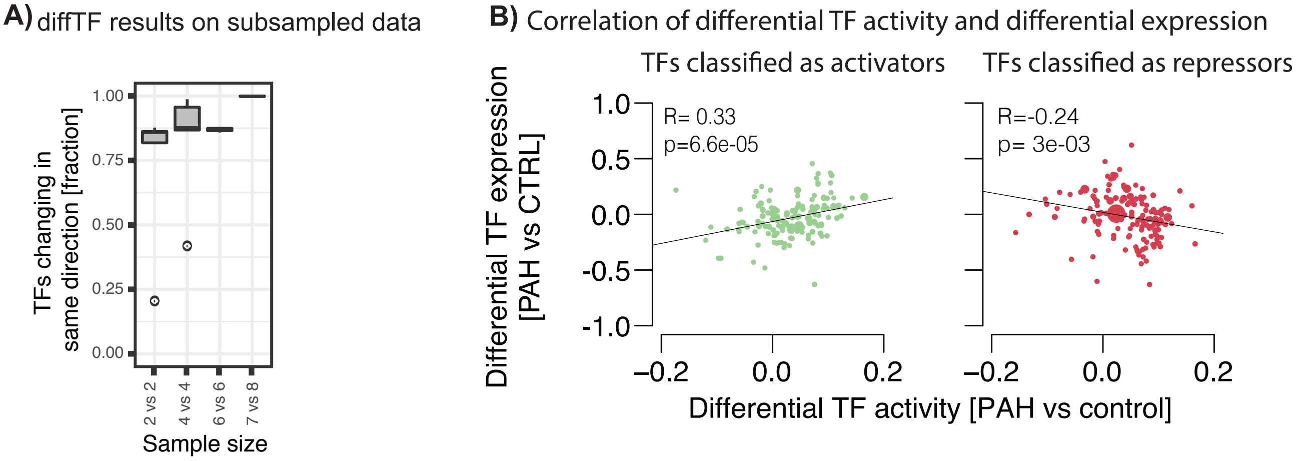

Supplementary Figure 3. Correlation of differential TF activity and differential TF expression. **A)** Scatterplot of differential TF activity vs differential expression for TFs classified as activators (left) or repressors (right). Classification was performed using diffTF in classification mode. **B)** Power analysis of the differential TF activity analysis**.** We chose a subset of samples to perform the diffTF analysis and quantified the fraction of TFs that were recovered as significantly differentially (p-value 0.001). Of the total 10 PAH plus 7 control samples we compared each 2 vs 2, 4 vs 4, 6 vs 6, 7 vs 8 choosing 5-7 runs for each comparison. The y-axis denotes the fraction of TFs that show a change in TF activity in the same direction as for the full 10 vs 7 data (at p-value <0.001). Note that for each permutation, the original design had to be adjusted to the chosen subsample by removing one or multiple variables that were originally in the design formula: batch, mutation, gender. This is particularly true for the 2 vs 2 group. The small variability for 8 vs 7 and 6 vs 6 can be explained by the fact that for the smaller group, either all (8 vs 7) or all except one (6 vs 6) control samples are taken, thereby not allowing much variability. The only variability then comes from the random selection of 6 / 8 samples from the PAH group. Boxplots: box indicates 25-75 percentile, whiskers 20/90 and outliers are shown as points.

Supplementary Figure 4

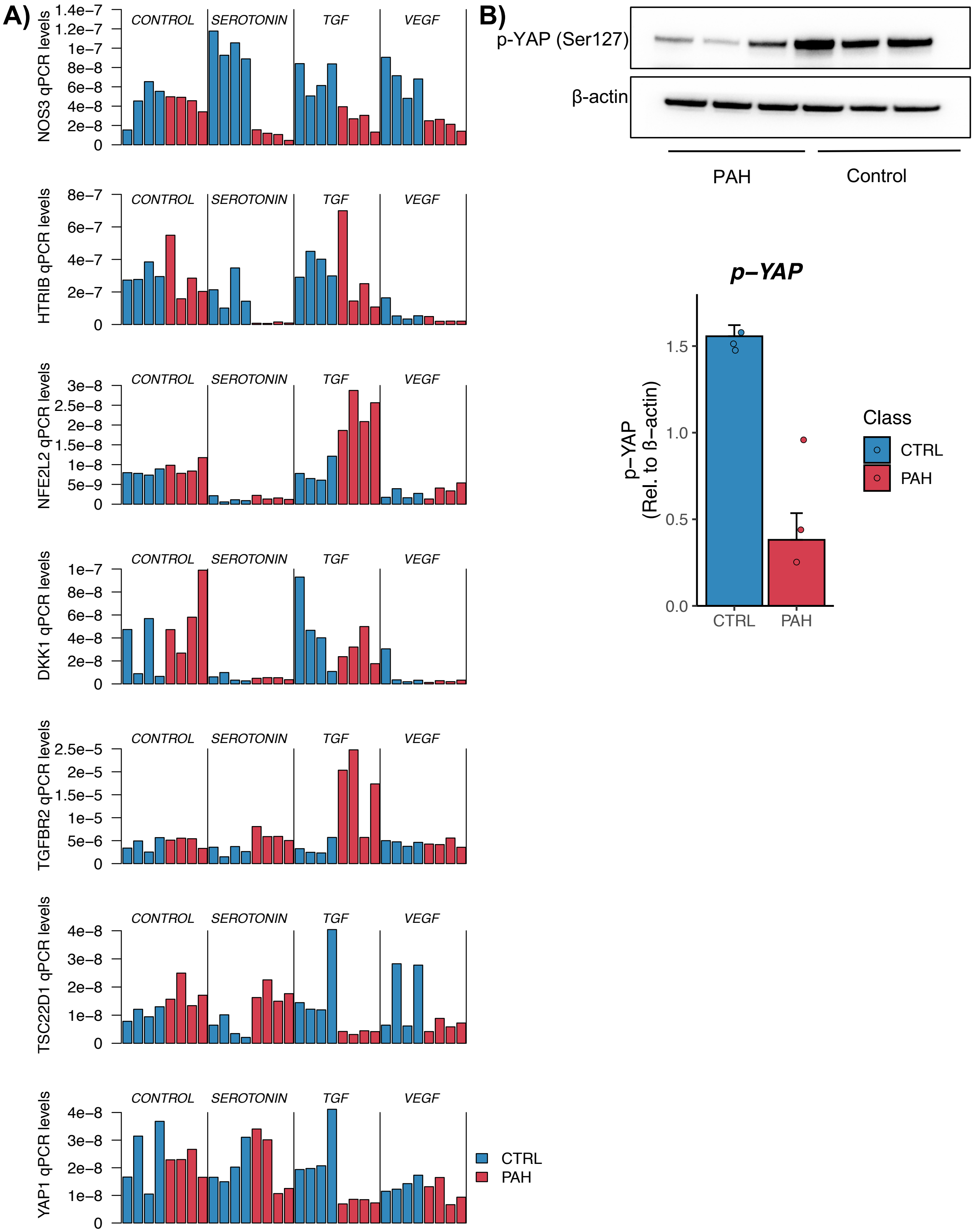

Supplementary Figure 4. A) RT-PCR levels for baseline and after a stimulation with endothelial-specific growth factors TGFβ, serotonin and VEGF on a selection of genes relevant for endothelial biology. **B)** Western blots of phosphorylated form of YAP1. **top:** Raw data of p-YAP at residue Ser127 is show for PAECs of PAH patients vs controls. Measurements were taken after stimulation with TGF-beta. **bottom:** Quantification of the signal relative to beta-actin, which served as internal control (n=3 patients, 3 controls). Error bars indicate standard deviation across three replicates. Source data are provided as a Source Data file.

4. Supplementary References

1. Yu, L., Fernandez, S. & Brock, G. Power analysis for RNA-Seq differential expression studies. *BMC Bioinformatics* **18,** 234 (2017).
2. Landt, S. G. *et al.* ChIP-seq guidelines and practices of the ENCODE and modENCODE consortia. *Genome Res.* **22,** 1813–1831 (2012).
