# Supplementary material for: Remodeling of active endothelial enhancers is associated with aberrant gene-regulatory networks in pulmonary arterial hypertension
Source: Nat Commun. 2020 Apr 3;11:1673. doi: 10.1038/s41467-020-15463-x (PMC7125148; doi:10.1038/s41467-020-15463-x)
Supplement: Supplementary file 4 — Description of Additional Supplementary Files [file 41467_2020_15463_MOESM4_ESM.pdf]

Description of Additional Supplementary Files

File Name: Supplementary Data 1
Description: Sample table of metadata and conducted assays in this study for patients of pulmonary arterial hypertension (PAH) and controls.

File Name: Supplementary Data 2
Description: Differentially modified loci for H3K27ac in pulmonary arterial endothelial cells (PAECs) from PAH-patients versus controls.

File Name: Supplementary Data 3
Description: Differentially active transcription factors based on genome-wide H3K27ac signal in PAECs from PAH-patients versus controls.

File Name: Supplementary Data 4
Description: Known-PAH associated genes and the source of information.

File Name: Supplementary Data 5
Description: PAH-specific gene regulatory network for pulmonary arterial endothelial cells.

File Name: Supplementary Data 6
Description: Enrichment-analysis of gene ontology annotations for genes within chromatin regulatory domains and targeted by differentially active transcription factors in the PAH-specific Gene Regulatory Network (only GO terms with an adj. p-value<0.05 were kept).
